# Non-compliant packaging and illicit smokeless tobacco in Bangladesh, India and Pakistan: findings of a pack analysis

**DOI:** 10.1136/tc-2021-057228

**Published:** 2022-09-27

**Authors:** S M Abdullah, Rumana Huque, Kamran Siddiqi, Mona Kanaan, Samina Huque, Safat Ullah, Suneela Garg, Mongjam Meghachandra Singh, Chetana Deshmukh, Amod L Borle, Romaina Iqbal, Laraib Mazhar, Mark Parascandola, Ravi Mehrotra, Ray Croucher, Zohaib Khan

**Affiliations:** 1 Health Sciences, University of York, York, UK; 2 Department of Economics, University of Dhaka, Dhaka, Dhaka District, Bangladesh; 3 Research and Development, ARK Foundation, Dhaka, Bangladesh; 4 Office of Research Innovation and Commercialization, Khyber Medical University, Pehsawar, Khyber Pakhtunkhwa, Pakistan; 5 Department of Community Medicine, Maulana Azad Medical College and Associated Hospitals, New Delhi, Delhi, India; 6 Department of Community Health Sciences, Aga Khan University, Karachi, Pakistan; 7 Department of Medicine, Aga Khan University, Karachi, Pakistan; 8 National Cancer Institute, Division of Cancer Control and Population Sciences, Bethesda, Maryland, USA; 9 Indian Council of Medical Research (ICMR) - Indian Cancer Research Consortium, New Delhi, Delhi, India

**Keywords:** illegal tobacco products, low/middle income country, packaging and Labelling, non-cigarette tobacco products, surveillance and monitoring

## Abstract

**Introduction:**

Illicit smokeless tobacco (ST) trade has seldom been documented despite ST use in at least 127 countries across the world. Based on non-compliance with packaging regulations, we report the proportion of illicit ST products from samples on sale in Bangladesh, India and Pakistan where 85% of global ST users reside.

**Methods:**

We purchased unique ST products from tobacco sellers in two purposively selected administrative areas (division/district) in each of the three countries. The criteria to determine illicit ST products were based on country-specific legal requirements for ST packaging and labelling. These requirements included: ‘market retail price disclosure’, ‘sale statement disclosure’, ‘pictorial health warning (PHW) pertinence’, ‘appropriate textual health warning’ and ‘using misleading descriptors (MDs)’. Non-compliance with even one of the legal requirements was considered to render the ST product illicit.

**Results:**

Almost all ST products bought in Bangladesh and India were non-compliant with the local packaging requirements and hence potentially illicit, all products in Pakistan lacked desirable features. The most common feature missing was health warnings: 84% packs in Bangladesh, 93% in India, and 100% in Pakistan either did not have PHW or their sizes were too small. In Bangladesh, 61% packs carried MDs. In India and Pakistan, the proportions of such packs were 32% and 42%, respectively.

**Conclusions:**

Weak and poorly enforced ST control policies may be slowing the progress of tobacco control in South Asia. Standardised regulations are required for packaging and labelling ST. Improving compliance and reducing sale of cheap illicit products may require business licensing and market surveillance.

**WHAT IS ALREADY KNOWN ON THIS TOPIC**

- Studies on illicit tobacco trade focus exclusively on cigarettes and are conducted mostly in high and upper middle-income countries. Illicit trade of smokeless tobacco (ST) products has seldom been a focus despite documented ST use in at least 127 countries across the world with over 350 million users.

**WHAT THIS STUDY ADDS**

- In the three countries where 85% of ST users reside, the majority of ST products are non-compliant with packaging regulations and hence potentially illicit.

**HOW THIS STUDY MIGHT AFFECT RESEARCH, PRACTICE OR POLICY**

- In Bangladesh, India and Pakistan, there is a need to implement and enforce effective standardised regulations for ST products.

## Introduction

Tobacco use leads to over 8 million deaths each year globally.^1^ Tobacco products and their use vary across different geographical regions. Besides smoked forms (eg, cigarette, bidi, waterpipe, cigar), smokeless tobacco (ST) products such as zarda, gul, khaini, sada pata (sun-dried tobacco leaf), naswar and gutka are also popular, particularly in Bangladesh, India and Pakistan—where 85% of the global ST users reside.^2^ According to the latest Global Adult Tobacco Survey in Bangladesh (2017), India (2017) and Pakistan (2014), the prevalence of ST use among adults was 21%,^3 4^ 21.4%,^4 5^ and 8.6%, respectively.^6^ The excessive ST use in these countries and its associated health risks require stringent measures for effective tobacco control.^7^ However, ST control has not been a priority policy focus to date.^8^ Low price, easy affordability and accessibility, social and cultural acceptance, misconception about its medicinal value, exposure at a young age and a lack of regulatory framework contribute to high prevalence of ST in these countries.^9–11^

Despite being signatories to the WHO Framework Convention for Tobacco Control (FCTC), the three countries differ in terms of regulations for ST products. The tobacco control laws in Pakistan are not comprehensive and exclude ST from most provisions. In India, the law requires pictorial health warning (PHW) labels to cover 85% of the principal display area of ST packs, while in Bangladesh such requirement is only to cover 50%. Both countries require the warnings to rotate but the time intervals are different. Printing misleading descriptors such as ‘light’ and ‘low tar’ on ST packs is prohibited by law in India and Bangladesh. In Bangladesh, the textual health warning (THW) must be printed in Bengali; in India, it can be printed in English or in an Indian language or both. Additionally, there are bans on some ST products, for example, gutka and pan masala are banned in India. Despite the ban, pan masala and gutka ingredients are on sale in separate small pouches and consumers can mix the ingredients to consume the products. In Bangladesh, no ST products are banned, while in Pakistan the manufacture of gutka is banned in Sindh, one of the country’s four provinces.

Tax evasion—termed loosely as illicit trade in this paper—can increase tobacco’s affordability by undercutting its price. Illicit trade increases tobacco consumption and erodes governments’ revenue, thus undermining tobacco control efforts.^12 13^ Most studies on illicit tobacco trade focus exclusively on cigarettes and are conducted mostly in high and upper middle-income countries.^12 14–28^ Illicit trade of ST products has seldom been a focus despite documented ST use in at least 127 countries across the world with over 350 million users.^29^ Apart from a handful of single-country reports on packaging compliance, advertising and promotion,^30–35^ multicountry studies comparing the nature and share of illicit ST sales are non-existent. The absence of any trace and track system or even tax stamps on ST products makes it impossible to estimate the share of illicit ST trade accurately.

This manuscript reports the extent of illicit ST products on sale defined on the basis of non-compliance with packaging laws in Bangladesh, India and Pakistan. In the absence of tax stamps, non-compliance with ST packaging and labelling requirements was used as a proxy for tax evasion.^27 36 37^ In previous studies estimating illicit cigarette trade, when packs did not comply with packaging laws, it was assumed that legal taxes have also not been paid.^27 37 38^ Where ST-specific regulations were absent, we estimated the proportion of products that would be non-compliant if the regulations were in place following WHO FCTC guidelines.

## Methodology

### Study design

The analysis presented in this paper is part of a broader mixed-methods study consisting of ST point-of-sale (POS) mapping and surveys, in-depth interviews with ST supply chain actors and ST products compliance with existing laws.^39^

### Study period and settings

The study was conducted in two purposively selected administrative areas (division/district) in each of the three countries: Dhaka and Rangpur districts in Bangladesh; North-East and North-West districts of Delhi in India; and Karachi and Peshawar in Pakistan. These were selected due to the high level of consumption and diversity of ST products in these areas. The data collection was completed between June 2019 and December 2020.

### Sampling

Within each participating district, one rural and one urban subdistrict was selected purposely. Country-specific definitions as per administrative documents and census reports were used to categorise the study subdistricts into urban and rural. In each of the subdistricts, three smaller areas (Union Council or Thana)—Primary Sampling Unit (PSU)—were randomly selected. Two enumeration blocks (villages or neighbourhood areas)—Secondary Sampling Unit (SSU)—were randomly selected within each PSU. All ST POS vendors (general/departmental stores, petrol pump/gas station stores, beer/liquor stores, grocery stores, betel quid shops, exclusive tobacco shops, discount shops, mobile vendors/carts, stationary carts) in each SSU were geomapped to construct a sampling frame. In case of geomapping mobile vendors/carts, the first contact place of the enumerators was considered as their location.^39^ Assuming 20% non-compliance in tobacco shops,^40–42^ 7% absolute precision, 5% confidence coefficient and 15% non-response, the optimum number of POS vendors in each country was estimated and rounded to be 290. As there were six enumeration blocks in each site, random selection of a maximum of 13 POS vendors from the sampling frame of each SSU resulted in a total maximum of 78 POS. Following that in every country both study sites had two strata (urban and rural), recruited number of POS could be as high as 312 in each of them.^39^ A thematic chart of the adopted multistage sampling method is presented in online supplemental figure 1.

10.1136/tc-2021-057228.supp1 Supplementary data

### Pack collection

Adapting Tobacco Packs Surveillance System (TPackSS) unique pack sampling process,^43^ ST sample packs were collected from the randomly selected POS vendors in each SSU. Based on their brand names, pack features (such as size, design, colour, cellophane, material), country of production, presentation, promotional message, text and pictorial warning, all available unique ST packaged products were purchased. At the first selected POS, all unique packs of locally available ST products were collected. This was followed by the collection of only those packs which were not procured previously, from the subsequent POS.

### Training of staff and pack characteristics

The collected sample packs were categorised according to the product type as identified by the WHO FCTC Global Knowledge Hub for ST.^44^ The hub categorised the ST products in terms of four parameters, namely, ‘ingredients used for preparation’, ‘modality of use’, ‘places or areas of use’ and ‘most commonly prevalent region/country/gender’.^39^ Data entry personnel were trained in using the Tobacco Advertisement and Promotion Survey pack analysis tool.^45^ The analysis involved recording the following information: ST product category, brand name, country of origin, manufacturer details (name and address), price, tax and ingredient disclosure (whether printed on the pack), pack form (paper box, sachet, plastic bottle, tin can, etc) and weight (measured in grams), presence and appropriateness of PHW and THW, culture-specific colour and symbol (eg, reference picture, flower, animal, place, etc) and/or statement of health claims (eg, mentioning product is less harmful, refreshing and safer, contains low level of harmful substance, etc). Our trained operators recorded ST pack information in a standardised database developed using Microsoft Access. The packs were double-coded and data were cross-checked for accuracy and consistency. The size of warning labels was measured after excluding the black borders. Given the irregular shapes of some ST packs, the face and flat portions were measured; for packs with bevelled and rounded edges, these bevelled and rounded portions were excluded. For the soft packs, the face portion was measured excluding the foil area. Further methodological details can be found in the TPackSS codebook.^46–49^

### Packaging non-compliance and defining illicit packs

To estimate the share of illicit ST on sale in study areas, we developed a definition of what constitutes ‘illicit’. We consulted relevant literature^13 50^ and the FCTC Article 11.1(a), 11.1(b) and Article 15.2(a)^51^ to identify the features that might be used as proxy for illicit tobacco products. Once core features (market retail price (MRP) disclosure, sale statement disclosure, tax stamp and banderols display, PHW pertinence, appropriate THW and misleading descriptors (MDs)) were agreed, these were mapped across the national laws in the three countries (online supplemental tables 1–3).^52–56^ The mapping revealed that the study countries did not have uniform regulations. In Pakistan, there were no regulations that could be applied to ST packs (table 1). The only rule that existed for ST products in Pakistan, categorised a product as illegal if it is imported and originated from India or Israel (online supplemental table 3). In Bangladesh, it was mandatory for ST to carry ‘MRP’ and ‘sale statement’ (printing ‘sales only allowed in (country’s name)’ on the pack). In contrast, India did not require a ‘sale statement’. While country-specific laws existed for PHW, THW, and MDs in Bangladesh and India, there were no requirements for affixing fiscal instruments (tax stamps or banderols) on ST packs (table 1).

10.1136/tc-2021-057228.supp2 Supplementary data

10.1136/tc-2021-057228.supp3 Supplementary data

10.1136/tc-2021-057228.supp4 Supplementary data

**Table 1:** Hallmarks and definitions of illicit ST packs as per country-specific rules

Pack features	Country-specific rule existence for ST and illicit hallmarks								
	Bangladesh			India			Pakistan		
	Rule status	Illicit hallmark	Comment	Rule status	Illicit hallmark	Comment	Rule status	Illicit hallmark	Comment
a. MRP disclosure	Yes	Yes	Operational definition of illicit ST packs in Bangladesh included 5 parameters defined under pack features (a), (c), (d), (e) and (f). An ST pack found non-compliant with even one of the parameters was considered illicit.	Yes	Yes	Operational definition of illicit ST packs in India included 4 parameters defined under pack features (a), (d), (e) and (f). An ST pack found non-compliant with even one of the parameters was considered illicit.	None	No	In the absence of country-specific rules in Pakistan, ST packs were not classified as illicit. We merely described the proportion of ST products under various categories.
b. Tax stamp and banderols display	None	No		No	No		None	No	
c. Sale statement disclosure	Yes	Yes		None	No		None	No	
d. Pictorial health warning (PHW)	Yes	Yes		Yes	Yes		None	No	
e. Textual health warning (THW)	Yes	Yes		Yes	Yes		None	No	
f. Misleading descriptors (MDs)	Yes	Yes		Yes	Yes		None	No	

‘Sale statement disclosure’ indicates printing ‘sales only allowed in (country name)’; MD includes the use of a culturally specific reference such as colour or symbol (such as star, flower, birds, tree, sun, misleading photograph, tar, low tar, mild, etc), use of words indicating flavour, and/or strength (such as super quality, fresh, smooth, special, royal, rose, etc), and affixing health claim (such as mentioning product is less harmful, refreshing and safer, contains low level of harmful substance, etc). In many cases, ST packs in Bangladesh and India contain photographs of ‘bride’, ‘monk’, a ‘Hindu God’, etc; in Pakistan pictures of ‘horse’, ‘star’, ‘tiger head’, etc. For PHW, the law in Bangladesh and India requires covering at least 50% and 85% of the display area, respectively. National laws in both these countries also require THW to be written in the principal language. The word ‘None’ indicates the non-existence of rule for ST products in the country’s tobacco control policy for the relevant pack features for regulation.

MRP, market retail price; ST, smokeless tobacco.

We used country regulations pertaining to ST packs to select the hallmarks of illicit packs. For Bangladesh and India, a product was considered illicit (unless otherwise stated) if it had any of the following: no MRP printed, no sale statement disclosure (only for Bangladesh), no and/or inappropriate proportion of PHW (Bangladesh–at least 50%, India–at least 85%), no and/or inappropriate THW (not written in principal language) and affixing MDs (using culturally specific reference such as colour or symbol and/or words suggesting flavour or strength and/or making health claims). In the absence of ST-specific regulations in Pakistan, we simply described the proportion of packs under different descriptions and did not classify these as illicit.

### Statistical analysis

All analyses were stratified by country. We presented frequencies and percentages to describe the features of packs, namely, type of ST products, whether pre-packaged by manufacturer, pack form and whether the name and address manufacturer was printed on the pack. For each compliance feature, we calculated frequencies and percentages of pack that were compliant. Based on country-specific defined criteria for illicit ST products, we computed the proportion of illicit products. Estimates of the proportion of illicit products were presented with 95% CI. The statistical analysis was carried out using STATA V.15.^57^

## Results

We examined the compliance of ST packs in study countries. In addition to each specific illicit hallmark, the overall proportions of illicit ST packs on sale among the total pack purchased were described accordingly.

### Sample description and compliance across countries

Table 2 describes ST packs and their features and compares their compliance with existing packaging and labelling laws across the three countries. We collected 116 unique ST packs (categorised broadly under zarda, gul and pan masala with tobacco) in Bangladesh. The sample for India was 41 (zarda, gul, khaini, naswar and pan masala with tobacco) and 64 for Pakistan (naswar, gutka, khaini, pan masala with tobacco, mawah and snus). In Pakistan, the samples were dominated by naswar (59.3%); whereas in India and Bangladesh, pan masala with tobacco (46.3%) and zarda (85.3%), respectively, were most common. Across the study countries, ST products were sold both pre-packed and loose (the latter are commonly mixed with other products such as betel leaf and betel nut). A considerable variation in the form of ST packs was observed across the countries. While in Bangladesh, a tin or a can (44.0%), plastic bottles (38.8%) or sachets (12.9%) were used to pack ST by their manufacturers, in India sachets were the predominant (80.4%) type of packaging. Paper packets or boxes (57.8%) and sachets (29.7%) were found to be the most popular form of packaging in Pakistan.

**Table 2:** Comparative analysis of ST pack compliance in Bangladesh, India and Pakistan

ST products and pack features	Bangladesh (%)	India (%)	Pakistan (%)
Type of ST products			
Zarda	99	10	–
Naswar	–	1	38
Gul	7	3	–
Gutka	–	–	9
Mawah	–	–	3
Snus	–	–	1
Khaini	–	8	8
Pan masala with tobacco	10	19	5
Packs (N)	116	41	64
Pre-packaged by manufacturer (yes)	113 (97.41)	41 (100.00)	55 (85.93)
Pack form			
Paper packet/box	5 (4.31)	5 (12.20)	37 (57.81)
Tin/can	51 (43.97)	3 (7.32)	8 (12.50)
Plastic bottle	45 (38.79)	–	–
Sachet	15 (12.93)	33 (80.49)	19 (29.68)
Name and address of manufacturer (yes)	93 (80.17)	35 (85.37)	33 (51.56)
Ingredient disclosure			
Ingredient listed (yes)	54 (46.55)	17 (41.46)	17 (26.56)
Weight per ingredient (yes)	4 (3.45)	3 (7.32)	4 (6.25)
Country of origin (yes)	107 (93.04)	32 (78.05)	29 (45.31)
Textual health warning (THW)			
THW status (yes)	93 (80.17)	40 (97.56)	13 (20.31)
Language of THW			
National/regional	81 (87.10)	38 (95.00)	1 (7.69)
Pictorial health warning (PHW)			
PHW status (any size) (yes)	90 (77.59)	40 (97.56)	–
Proportion of PHW			
25%–49%	72 (80.00)	5 (12.19)	–
50%–74%	15 (16.67)	14 (34.14)	–
75%–84%	3 (3.33)	18 (43.90)	–
85% and above	–	4 (9.75)	–
Provision of quit information (yes)	–	3 (7.32)	–
Affixing culturally specific reference (yes)	36 (31.03)	6 (14.63)	24 (37.50)
Words suggesting flavour or reduced strength (yes)	13 (11.20)	8 (19.51)	12 (18.75)
Statement of health claim (yes)	36 (31.03)	3 (7.32)	9 (14.06)

Numbers presented are counts and those in parenthesis are percentages.

ST, smokeless tobacco.

Manufacturer details were missing from 50% of the samples in Pakistan, 20% in Bangladesh and 15% in India. Similarly, 55%, 22%, and 7% of products in Pakistan, India, and Bangladesh, respectively, had no written information on the country of origin. With regard to ingredients disclosure on ST packs, there were marked differences across the countries. In Bangladesh and India, more than half of the ST packs had no information about the ingredients. Among the packs where ingredients were listed, about 97% in Bangladesh and 93% in India had no information regarding the exact weight per ingredient the manufacturer had used for the product. Only around 27% of the ST packs in Pakistan displayed ingredient lists; however, 94% of these lists did not mention their weights.

Considering THW and PHW, in India only 2% of ST packs had no THW. Among those that contained THW, 95% used national or regional languages. On the other hand, around 20% of packs had no THW in Bangladesh and 80% had no THW in Pakistan. Around 87% of ST pack warnings in Bangladesh used the national language but only 8% did so in Pakistan. Similar observations were made for PHW. Although the majority of ST packs contained PHW in India, 9 out of 10 PHWs were not compliant with the required size (minimum 85% of pack surface). In Bangladesh, 22% of packs had no PHW and 8 out of 10 did not meet the size requirements (minimum 50% of pack surface). None of the packs in Pakistan contained PHW.

None of the ST packs collected in Bangladesh and Pakistan and very few in India (7%) had any quit information. Affixing culturally specific references to attract the respective consumers appeared to be a common practice among the ST manufacturers in the study countries. Among ST packs in Bangladesh, 31% used statements of health claims. About 20% of the ST packs in India contain MDs of flavour or strength.

### Packaging non-compliance and percentage of illicit ST packs sales

Alongside the overall proportion of illicit ST packs on sale, table 3 summarises the proportion of individual hallmarks considered in the definition of illicit. Although required by law, around 57% of ST packs did not have MRP affixed in Bangladesh. Regarding the health warnings, approximately 84% of the packs had either no PHW or inappropriate size of PHW. Another 30% did not have THW or used unprescribed language. Sixty-one per cent of the packs used MDs such as culturally specific reference, words indicating flavour and/or strength, and health claims and regarded as illicit. Sale statement disclosure was absent on 72% of packs. Among 116 packs in Bangladesh, 107 were non-compliant and hence the share of illicit ST products was estimated as 92%. In India, the share of illegal products in the market concerning specific features such as MRP (2.4%), THW (7.3%) and MDs (31.1%) was considerably lower. Nevertheless, 38 out of 41 ST packs were non-compliant with regard to PHW, making 93% of these illicit. In Pakistan, all 64 products had sale statement disclosure absent and missing or inappropriate PHW, all except one had missing THW and 42% packs had MDs. Thus, in Pakistan, all ST packs found had improper features.

**Table 3:** Packaging non-compliance and percentage of illicit ST products in Bangladesh and India, and the percentage ST products with specific pack features in Pakistan

Pack features (themes for illicit ST products)	Bangladesh		India		Pakistan	
	Non-compliant packs	Illicit percentage (95% CI)	Non-compliant packs	Illicit percentage (95% CI)	Packs with features	Percentage of packs with specific features (95% CI)
(a) MRP not printed	66	56.9 (47.4 to 66.1)	1	2.4 (0.10 to 12.9)	–	–
(c) No sale statement disclosure	84	72.4 (63.6 to 80.4)	–	–	64	100 (94.4 to 100.0)*
(d) No PHW or inappropriate size of PHW (Bangladesh and Pakistan–below 50%, India–below 85%)	98	84.4 (76.6 to 90.5)	38	92.6 (80.1 to 98.5)	64	100 (94.4 to 100.0)*
(e) No THW or inappropriate language of THW (not in principal language)	35	30.1 (22.0 to 39.4)	3	7.3 (1.5 to 19.9)	63	98.4 (91.6 to 100.0)
(f) Presence of any misleading descriptors (culturally specific reference or words indicating flavour and/or strength or health claim)	71	61.2 (51.7 to 70.1)	13	31.7 (18.1 to 48.1)	27	42.1 (29.9 to 55.2)
Overall estimate of non-compliant and hence illicit ST packs (in Bangladesh, % of ST packs have at least one of the attributes listed from (a) to (f); in India, % of ST packs have at least one of the attributes listed in (a), (d), (e) and (f); and in Pakistan (packs with specific features), % of ST packs have at least one of the attributes listed from (c) to (f)).	107	92.2 (85.8 to 96.4)	38	92.6 (80.1 to 98.5)	64	100 (94.4 to 100.0)*

*Indicates one-sided 97.5% CI. In the absence of country-specific rules in Pakistan, ST packs were not classified as illicit. We merely described the proportion of ST products under various categories.

MRP, market retail price; PHW, pictorial health warning; ST, smokeless tobacco; THW, textual health warning.

## Discussion

We compared ST pack compliance with national laws in Bangladesh, India and Pakistan, and almost all ST products were found non-compliant and potentially illicit in Bangladesh and India. In the absence of ST-specific laws in Pakistan, compliance could not be assessed; however, as expected, none were found in accordance with the FCTC guidelines. In 2016, non-compliance with regard to PHW among zarda and gul in Bangladesh was found to be 40% and 24%, respectively^32^; a previous study in India reported only 2% of the packs as compliant with warning size^58^ and none of the ST packs had PHW in Pakistan.^9^ The effectiveness of appropriate health warning labels in knowledge enhancement, quit intention and youth tobacco uptake is well recognised.^59–63^ However, the ST market regulation and enforcement of PHW on ST packs remain ineffective despite these countries being WHO FCTC signatories.

A further area of concern about ST pack non-compliance is related to THW. Bangladesh and Pakistan, respectively, had 53% and 68% of ST packs carrying THWs^9^; while in India, about 93% of the packs were compliant, considering the language as an indicator.^58^ Although in Bangladesh THW compliance has improved, around 30% of packs still have no THW or have them in inappropriate language. In India, language compliance has remained more or less stable having only 7% non-compliance packs. As expected, the situation has not improved in Pakistan and 98% of packs had no THW.^9^ Three-fifths of the packs (61%) used MDs in Bangladesh and were categorised as illicit. In India and Pakistan, the share of such packs was 32% and 42%, respectively. This is an urgent policy concern as ST products are usually low-cost tobacco options and consumption is prevalent largely in rural areas, low-income classes and people with low educational attainment.^64^ Illusive packaging content may undermine the national tobacco control effort by attracting this population.

The absence of ST-specific policies in Pakistan meant that international evidence-based practices outlined in WHO FCTC guidelines are not in place. In Bangladesh and India although regulations are in place, their poor implementation, for example, size of PHW or use of MDs, has weakened their impact. Additionally, formal guidelines are yet to be adopted for certain WHO FCTC articles. In the absence of formal guidelines, many WHO FCTC articles have not been implemented comprehensively.^65^ In contrast, market monitoring and policy surveillance are active for smoking tobacco products. Consequently, their compliance is relatively better in the three countries.^32 66 67^ Recent studies reveal that share of illicit cigarette sale in India is 2.7%,^13^ in Bangladesh around 2%^68^ and in Pakistan 17.8%.^69^ Though Bangladesh, India and Pakistan ratified WHO FCTC for comprehensive tobacco control, ST policies and their enforcement mechanisms remained weak; taxes are low and poorly administered; and in general, the ST control policies, where in place, are inadequate.^9 70^

The supply chain and market for ST are distinctive with many fragmented and informal supply chain actors. ST production and consumption are culturally accepted in South Asia.^9 35^ The manufacturers of ST products are diverse. Across the three study countries, besides a few influential and enormous companies (eg, Dharmpal Satyapal, Som Sugandh Industries and Dhariwal Industries in India, and Kaus Chemical Works, Baba Al-Tajer Dhaka, etc in Bangladesh), the ST producers are largely home based and work as unregistered small entrepreneurs with informal establishments.^70^ Stable and high demand, small start-up capital requirement, and low risk with quick, good income play as an incentive for such business.^9^ There is no standardisation of packs against enormous brands and product diversity for ST. These features, on the other hand, made the ST market fragmented.

The study countries are already overburdened with ST-related diseases.^29^ Any practices that make the ST products illicit and inexpensive will undermine tobacco control initiatives in these countries. Hence, the market and ST supply chain should be integrated within the regular tobacco supply chain and formalised. Standardised rules and practices needed to be in place for its manufacturing and packaging. Integration and emphasis for ST in tobacco control laws, country contextualisation of the policies, and effective monitoring, surveillance, and implementation should be a priority. Licensing requirements for manufacturing, marketing and distribution of ST products, and effective tracking and tracing of ST products should be in place. Essentially, the ST businesses should exist within a legal framework that is easy to administer.

This multicountry study is one of the very few attempts which aimed to assess ST pack compliance with the legal requirements. Besides the general pack compliance appraisal, it would help the policymakers to understand the nature and extent of illicit products in countries with high ST-related disease burden. It re-emphasised the poor regulation problem of the ST market employing rigorous research methods and standardised tools.

Since the results are obtained from only two purposively selected administrative areas in each study country, these cannot be generalised to the whole country and the scope of study remains limited. Nevertheless, the basis of purposive selection was the extent of use, size, and diversity of the population and variety of ST products. Moreover, all PSUs and SSUs were randomly selected, increasing the validity of findings. As the analysis relied only on packs and considered their compliance, differentiation between counterfeit and smuggled products was not possible. Although a standardised method (TPackSS)^38^ for analysing the ST packs was followed, multiple pre-analysis calibration workshops for the data retrievers and double-coding and cross-checking of the data by independent researchers took place. However, there remains a remote possibility of misclassification, owing to diverse shapes, sizes and specifications of health warning labels and descriptors on ST packs. Also, THW compliance was assessed only in relation to language and did not include the other specifications such as colour and font coding, and recommended text warnings contemporaneous to the specific PHW. Further research is needed in this regard. While the ST mobile vendors or carts were considered in the sampling frame, in many cases products were not collected because of being unable to trace the vendors. Tracking the overtime trend of illicit share requires real-time information generation with multiple rounds of seller surveys based on pack collection. In addition, capacity strengthening of relevant authorities for tracking and tracing, and improving the enforcement is needed.

## Conclusions

We found that in Bangladesh, India and Pakistan, either evidence-based policies for ST control are not in place or their enforcement is weak to make any impact. Improving compliance and reducing cheap illegal products may require formalising ST market through business licensing and operational market monitoring. Prioritising ST in policy formulation and implementation should also be considered in this regard.

## Data Availability

Data are available upon reasonable request. Data are available upon reasonable request after completion of all intended publications from the study. The link to the published protocol is https://bmjopen.bmj.com/content/10/6/e036468. In this regard, the study chief investigator can be communicated (Siddiqi, Kamran, email: kamran.siddiqi@ york.ac.uk, ORCID iD: 0000-0003-1529-7778) for further queries.
