# Supplementary figures and images for: Non-compliant packaging and illicit smokeless tobacco in Bangladesh, India and Pakistan: findings of a pack analysis
Source: Tob Control. 2022 Sep 27;33(3):333–40. doi: 10.1136/tc-2021-057228 (PMC11041550; doi:10.1136/tc-2021-057228)

Figure S1: Multistage Sampling Design for Study Countries

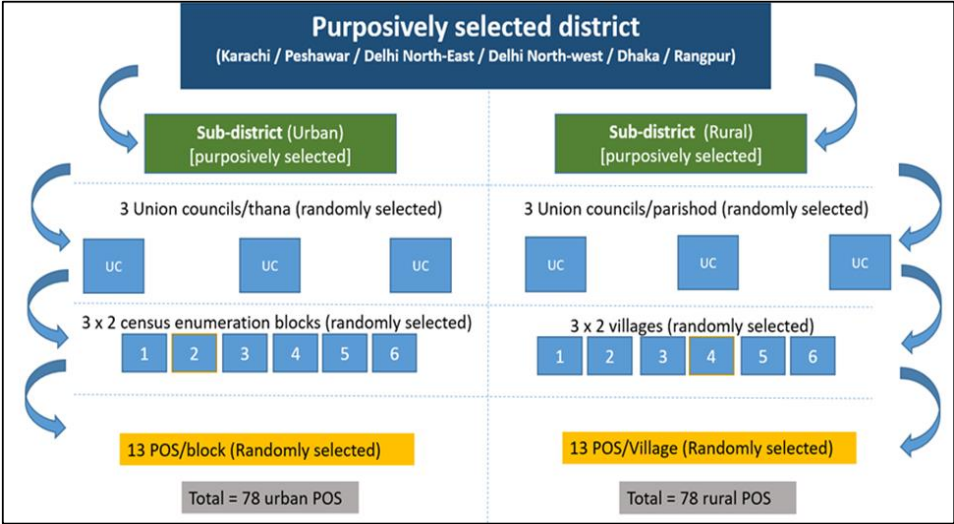

Source: Adapted from Zohaib et al. (2020)

Supplement: Supplementary data [file tc-2021-057228supp001.pdf]
