# Supplementary material for: Non-compliant packaging and illicit smokeless tobacco in Bangladesh, India and Pakistan: findings of a pack analysis
Source: Tob Control. 2022 Sep 27;33(3):333–40. doi: 10.1136/tc-2021-057228 (PMC11041550; doi:10.1136/tc-2021-057228)
Supplement: Supplementary data [file tc-2021-057228supp002.pdf]

Table S1: Tobacco Packaging Compliance Rules in Bangladesh against FCTC: Comparison of Smoking and Smokeless Tobacco

Pack Feature	FCTC Compliance Requirement(s) and Recommendations (Article 11 and Article 15)	Compliance Indicator(s)/Rule(s) (As per Bangladesh Law)	Smokeless Tobacco	Smoking Tobacco
**Price Disclosure**	No Specific Requirement	Mandatory to print the retail price (MRP) of the goods 'on the body of the goods or on every package, sachets or cells distinctly, conspicuously and indelibly'.	Applicable	Applicable
**Tax Stamp and Banderole**	No Specific Requirement	Government has made it obligatory to use tax stamp or banderole on Cigarette and Bidi packets.<br><br>Hard and Soft Pack Tax Stamp Specification:<br>Prime Color: Sky Blue, Pink, Light Green and Light Yellow (Different Tax Stamp Color for Different Price Tier)<br>Size: Length and Width – 45mm* 20mm<br>Shell and Slide Pack:<br>Prime Color: Light Blue and Pink<br><br>Size: Length and Width – 140±0.5mm* 14±0.25mm	No Specific Rule	Applicable
**Pictorial Health Warning (PHW)**	1.Front and Back of Principal Display Area (PDA)<br>2. Top of PDA<br>3. Opening does not damage/ conceal Health Warning<br>Recommendation:<br>1. Warnings not obstructed by other markings	**PHW Placement:**<br>1.Health warnings shall be printed on both sides of the mian display of packet, cover, carton or box of tobacco products. If the packets do not have two main sides in that case health warnings shall be printed in the upper part on the main display area.<br>2.Health warnings shall be printed in such a way as not to be covered up by attachment of stamp or band roll or for any other reason.	Applicable	Applicable
	1. Full color pictorial HW	**PHW Element (e.g. Color):** The color pictures and health warning messages provided by the government shall be printed as it is with the size, color, ratio etc. of the script	Applicable (NTCC Recommended)	Applicable (NTCC Recommended)
	1.50% or more but no less than 30% of the PDA<br>2.Text of HW bold, legible font size, style/color enhancing visibility and legibility	**PHW Size:** At least 50% of the total area of each main display area.	Applicable (NTCC Recommended)	Applicable (NTCC Recommended)
		**THW Placement:** Below the PHW	Applicable	Applicable

**Textual Health Warning (THW)**	1.Contrasting colors for background of text for text-based elements of warning<br>2. HW message addresses different issues related to tobacco use, in addition to harmful health effects (e.g., cessation, addictiveness, etc.)<br>Recommendations:<br>1. Innovative messages (e.g., outcomes on environment, industry practices)	**THW Element (Color and Statement):** The script shall be in white letters against a black background	(a)Smoking causes throat and lung cancer;<br>(b)Smoking causes respiratory problems;<br>(c)Smoking causes stroke;<br>(d)Smoking causes heart disease;<br>(e)Second-hand smoke causes harms to the fetus;<br>(f)Smoking causes harms to the fetus;<br>(g)Second – hand smoking causes death	(a) Consumption of tobacco products causes mouth and throat cancer;<br>(b) Consumption of tobacco products causes harms to the fetus.
		**THW Size:** Ratio of image to text is 6:1 (Font Size 18)	Applicable	Applicable
	1.HW appear in the principal language or languages	**THW Language:** Bengali	Applicable	Applicable
**Statement of Sale**	No Specific Requirement	All packets, covers, cartons and boxes sold in Bangladesh shall carry the statement: “Sales allowed only in Bangladesh”	Applicable	Applicable
**Misleading Descriptors**	1.Packaging must not promote terms, descriptors, signs that create false impression that product is less Harmful than others.<br>2. Prohibit display of figures for emission yields<br>3. Prevent display of expiry dates<br>The FCTC requires the Parties to take measures, within 3 years of the entry into force of the convention, to ensure that tobacco packages do not give misleading descriptions, such as "low tar", "ultra light", "mild" etc.	Packets, cartons, boxes, or covers of tobacco products shall not use brand elements (such as: light, mild, low-tar, extra, ultra, etc.) to create false impression about its impact and risk on public health.	Applicable	Applicable

Reference:

1. WHO. *WHO Framework Convention on Tobacco Control*. World Health Organization 2003.
2. National Board of Revenue. Ministry of Finance, Bangladesh. https://nbr.gov.bd/regulations/acts/vat-acts/eng (accessed 9 September, 2020).
3. National Tobacco Control Cell. Ministry of Health and Family Welfare, Bangladesh. https://ntcc.gov.bd/page/act-rules (accessed 9 September, 2020).
