# Supplementary material for: Non-compliant packaging and illicit smokeless tobacco in Bangladesh, India and Pakistan: findings of a pack analysis
Source: Tob Control. 2022 Sep 27;33(3):333–40. doi: 10.1136/tc-2021-057228 (PMC11041550; doi:10.1136/tc-2021-057228)
Supplement: Supplementary data [file tc-2021-057228supp003.pdf]

Table S2: Tobacco Packaging Compliance Rules in India against FCTC: Comparison of Smoking and Smokeless Tobacco

Pack Feature	FCTC Compliance Requirement(s) and Recommendations (Article 11 and Article 15)	COTPA/ Indian Finance Act	Smokeless Tobacco	Smoking Tobacco
**Price Disclosure**	No Specific Requirement	GST (Goods and Service Tax), Compensation Cess and NCCD (National Calamity Contingency Duty) are applicable on tobacco products.<br>The GST council fixed a statutory (exclusive) *ad-valorem* GST rate of 28% on all tobacco products with an additional compensation cess on cigarettes and SLT. This cess is also applied on value added at every stage of the supply chain, along with the GST.	Applicable	Applicable
**Tax Stamp and Banderole**	No Specific Requirement	Not compulsory in India	Not Applicable	Not Applicable
**Pictorial Health Warning (PHW)**	1.Front and Back of Principal Display Area (PDA)<br>2. Top of PDA<br>3. Opening does not damage/ conceal Health Warning<br>Recommendation:<br>1. Warnings not obstructed by other markings	Section 7-8 COTPA: Pictorial and Textual Health Warnings on Tobacco packs<br>1. The specified health warnings shall cover at least eighty-five per cent (85%) of the principal display area of the package of which sixty per cent (60%) shall cover pictorial health warning.<br>2. It should be made sure that none of the elements of the specified warning are severed, covered or hidden in any manner when the package is sealed or opened<br>3. No messages, images or pictures that directly or indirectly promote the use or consumption of a specific tobacco brand or tobacco usage in general or any matter or statement which is inconsistent with, or detracts from, the specified health warning are inscribed on the tobacco product package.<br>4. No product shall be sold unless the package contains the specified health warning.<br>Pictorial Health Warning(PHW) Placement:	Applicable	Applicable

		A pictorial representation of the ill effects of tobacco use on health shall be placed above the textual health warning.		
	1. Full color pictorial HW	PHW Element (e.g. Color): It shall be printed with four colours with printing resolution of minimum 300 DPI (Dots per inch).	Applicable	Applicable
	1.50% or more but no less than 30% of the PDA<br>2.Text of HW bold, legible font size, style/color enhancing visibility and legibility	PHW Size: The size of the specified health warning on each panel of the tobacco package shall not be less than 3.5 cm (width) x 4 cm (height), so as to ensure that the warning is legible, prominent and conspicuous.	Applicable	Applicable
**Textual Health Warning (THW)**	1.Contrasting colors for background of text for text-based elements of warning<br>2. HW message addresses different issues related to tobacco use, in addition to harmful health effects (e.g., cessation, addictiveness, etc.)<br>Recommendations:<br>1. Innovative messages (e.g., outcomes on environment, industry practices)	Textual Health Warning (THW) Placement: textual health warning and shall be positioned on the top edge of the package and in the same direction as the information on the principal display area	Applicable	Applicable
		1. Twenty-five per cent (25%) shall cover textual health warning and shall be positioned on the top edge of the package and in the same direction as the information on the principal display area<br>2. The textual health warning shall be inscribed in the language used on the pack<br>Each health warning shall be specified in English, Hindi and any other regional languages.<br>Appropriate language combination shall be selected to ensure that the language selected for health warning is in conformity with the language used on the package by the manufacturer or importer or packer.<br>Provided that where the language used on a package or on its label is :-<br>(a) English, the health warning shall be expressed in English<br>(b) English and Indian languages, the health warning shall be expressed in English and any one of the Indian languages in which the brand name appears;<br>(c) Hindi and other Indian languages, the health warning shall be expressed in Hindi and any one of	For smokeless forms tobacco products, the word warning shall appear in white font colour on a red background and words “tobacco causes mouth cancer” and “tobacco kills” shall appear in white font colour on a black background.	For smoking forms tobacco products, the word warning shall appear in white font colour on a red background and words “smoking causes throat cancer” and “smoking kills” shall appear in white font colour on a black background.

		the Indian language in which the brand name appears;<br>(d) Any Indian language, the health warning shall be expressed in such Indian language;<br>(e) Indian languages, the health warning shall be expressed in any two Indian languages in which the brand name appears;<br>(f) Foreign language, the health warning shall be expressed in English;<br>(g) Foreign and Indian languages, the health warning shall be expressed in English and any one of the Indian languages in which the brand name appears<br><br>The textual health warning shall appear in not more than two languages used on the package<br><br>For smoking and smokeless forms of tobacco products, the words 'TOBACCO CAUSES PAINFUL DEATH' shall appear in white font colour on a red background and the words 'QUIT TODAY CALL 1800-11-2356' shall appear in white font colour on a black background. The intensity of colour in the background of the textual health warning shall be: White: Cyan(C):0%, Magenta(M):0%, Yellow(Y):0% Key (K):0%, Red: C:0%, M:100%, Y:100% K: 0% and Black: C:0%, M:0%, Y:0% K: 100% (Colour conversion codes). The textual health warnings shall be printed with four colours with printing resolution of minimum 300 DPI (Dots per inch).[2.]		
		THW Size: Ratio of image to text is 6:1 (Font Size 18)	Applicable	Applicable
	1.HW appear in the principal language or languages	THW Language: Textual health warning shall be inscribed in the language used on the pack	Applicable	Applicable

		Provided that where the language used on a package or on its label is :-<br><br>(a) English, the health warning shall be expressed in English<br>(b) English and Indian languages, the health warning shall be expressed in English and any one of the Indian languages in which the brand name appears;<br>(c) Hindi and other Indian languages, the health warning shall be expressed in Hindi and any one of the Indian language in which the brand name appears;<br>(d) Any Indian language, the health warning shall be expressed in such Indian language;<br>(e) Indian languages, the health warning shall be expressed in any two Indian languages in which the brand name appears;<br>(f) Foreign language, the health warning shall be expressed in English;<br>(g) Foreign and Indian languages, the health warning shall be expressed in English and any one of the Indian languages in which the brand name appears		
**Statement of Sale**	No Specific Requirement	Not applicable in India	Not Applicable	Not Applicable
**Misleading Descriptors**	1.Packaging must not promote terms, descriptors, signs that create false impression that product is less Harmful than others.<br>2. Prohibit display of figures for emission yields<br>3. Prevent display of expiry dates<br>The FCTC requires the Parties to take measures, within 3 years of the entry into force of the convention, to ensure that tobacco packages do not give misleading descriptions, such as "low tar", "ultra light", "mild" etc.	No tobacco product package or label shall contain any information that is false, misleading, or deceptive, or that is likely or intended to create an erroneous impression about the characteristics, health effects, or health or other hazards of the tobacco product or its emissions. This prohibition includes, but is not limited to, the use of words or descriptors, whether or not part of the brand name, such as "light", "ultra light", "mild", "ultra mild", "low tar", "slim", "safer", or similar words or descriptors; any graphics associated with, or likely	Applicable	Applicable

		or intended to be associated with, such words or descriptors; and any product package design characteristics, associated with, likely or intended to be associated with, such descriptors.		

Reference:

1. WHO. *WHO Framework Convention on Tobacco Control*. World Health Organization 2003.
2. National Tobacco Control Programme. Ministry of Health and Family Welfare, India. https://main.mohfw.gov.in/major-programmes/other-national-health-programmes/national-tobacco-control-programme-ntcp (accessed 10 October, 2020).
3. John RM, Dauchy E, Goodchild M. Estimated impact of the GST on tobacco products in India. *Tob Control* 2019;**28**:506–12.
