# Supplementary material for: Non-compliant packaging and illicit smokeless tobacco in Bangladesh, India and Pakistan: findings of a pack analysis
Source: Tob Control. 2022 Sep 27;33(3):333–40. doi: 10.1136/tc-2021-057228 (PMC11041550; doi:10.1136/tc-2021-057228)
Supplement: Supplementary data [file tc-2021-057228supp004.pdf]

Table S3: Tobacco Packaging Compliance Rules in Pakistan against FCTC: Comparison of Smoking and Smokeless Tobacco

**Pack Feature**	**FCTC Compliance Requirement(s) and Recommendations (Article 11 and Article 15)**	**FCTC Compliance Requirement(s) and Recommendations (Article 11 and Article 15)**	**Compliance Indicator(s)/Rule(s) (As per Pakistan's Law)**	**Smokeless Tobacco**	Smoking Tobacco
**Price Disclosure**	No Specific Requirement	No Specific Requirement	Not Applicable	Not Applicable	Applicable
**Tax Stamp and Banderole**	No Specific Requirement	No Specific Requirement	Not Applicable	Not Applicable	Not Applicable
**Pictorial Health Warning (PHW)**	1.Front and Back of Principal Display Area (PDA)<br>2. Top of PDA<br>3. Opening does not damage/ conceal Health Warning<br>Recommendation:<br>1. Warnings not obstructed by other markings	1.Front and Back of Principal Display Area (PDA)<br>2. Top of PDA<br>3. Opening does not damage/ conceal Health Warning<br>Recommendation:<br>1. Warnings not obstructed by other markings	The picture and warning must be placed on the front (top) of the pack in Urdu and on the back (top) of the pack in English. Opening does not damage/ conceal Health Warning	Not Applicable	Applicable
	1. Full color pictorial HW	1. Full color pictorial HW	PHW Element (e.g. Color): The color pictures and health warning messages provided by the government shall be printed as it is with the size, color, ratio etc. of the script. Every year government will provide a new message for PHW to be printed on packs and will be responsible for rotation of messages	Not Applicable	Applicable
	1.50% or more but no less than 30% of the PDA<br>2.Text of HW bold, legible font size, style/color enhancing visibility and legibility	1.50% or more but no less than 30% of the PDA<br>2.Text of HW bold, legible font size, style/color enhancing visibility and legibility	PHW Size:<br>85% of the area should be covered by PHW in phase manner.<br><br>At least 50% of the total area of each main display area applicable from July from 2018<br><br>At least 60% of the total area of each main display area applicable from July from 2019.	Not Applicable	Applicable

**Textual Health Warning (THW)**			THW Placement: Below the PHW	Not Applicable	Applicable
	1.Contrasting colors for background of text for text-based elements of warning<br>2. HW message addresses different issues related to tobacco use, in addition to harmful health effects (e.g., cessation, addictiveness, etc.)<br>Recommendations:<br>1. Innovative messages (e.g., outcomes on environment, industry practices)	1.Contrasting colors for background of text for text-based elements of warning<br>2. HW message addresses different issues related to tobacco use, in addition to harmful health effects (e.g., cessation, addictiveness, etc.)<br>Recommendations:<br>1. Innovative messages (e.g., outcomes on environment, industry practices)	THW Element (Color and Statement): black on white background	Not Applicable	Applicable
			THW Size: 2mm text size	Not Applicable	Applicable
	1.HW appear in the principal language or languages	1.HW appear in the principal language or languages	THW Language:<br>Urdu on front<br>English on the back	Not Applicable	Applicable
**Statement of Sale**	No Specific Requirement	No Specific Requirement	All packets, covers, cartons and boxes sold in Pakistan shall carry the statement: "Sales allowed only in Pakistan"	Not Applicable	Applicable
**Misleading Descriptors**	1.Packaging must not promote terms, descriptors, signs that create false impression that product is less Harmful than others.<br>2. Prohibit display of figures for emission yields<br>3. Prevent display of expiry dates<br>The FCTC requires the Parties to take measures, within 3 years of the entry into force of the convention, to ensure that tobacco packages do not give misleading descriptions, such as "low tar", "ultra light", "mild" etc.	1.Packaging must not promote terms, descriptors, signs that create false impression that product is less<br>2.Harmful than others<br>3. Prohibit display of figures for emission yields<br>4. Prevent display of expiry dates	No messages, images or pictures that directly or indirectly promote the use or consumption of a specific tobacco brand or cigarette usage	Not Applicable	Applicable
	The only rule that existed for ST products in Pakistan, categorised a product as illegal if it is imported and originated from India or Israel (Import Policy Order 2020)				

Reference:

1. WHO. *WHO Framework Convention on Tobacco Control*. World Health Organization 2003.
2. Federal Board of Revenue. Federal Board of Revenue, Pakistan. https://www.fbr.gov.pk (accessed 15 October, 2020).
3. Cell TC. Ministry of National Health Services, Regulations and Coordinations, Pakistan. http://www.tcc.gov.pk/ (accessed 15 October, 2020).
4. Import Policy Order 2020, Ministry of Commerce, Government of Pakistan, https://www.commerce.gov.pk/wp-content/uploads/2020/09/Import-Policy-Order-25-09-2020.pdf (accessed 15 October, 2020)
